# Extensive Delayed Brain Atrophy after Resuscitation in a Patient with Multiple System Atrophy

**DOI:** 10.3389/fneur.2017.00754

**Published:** 2018-01-15

**Authors:** Sazuku Nisitani, Hirofumi Miyoshi, Yoji Katsuoka

**Affiliations:** ^1^Port Island Hospital, Kobe, Japan

**Keywords:** multiple system atrophy, hypoxic encephalopathy, brain atrophy, magnetic resonance imaging, resuscitation, alpha-synuclein

## Abstract

Brain magnetic resonance imaging (MRI) of multiple system atrophy (MSA) shows atrophy in the cerebrum, cerebellum, and brainstem. It is also characterized by specific patterns such as hyperintense lateral putaminal rim. MRI of hypoxic encephalopathy shows atrophy mainly in the gray matter, and laminar necrosis in the cerebral cortex is often observed. Here, we report an MSA patient damaged by hypoxic insult and resuscitated after 18-min cardiac arrest. The brain of the patient developed severe atrophy within a period of 10 months. Furthermore, brain atrophy was observed in the white and gray matter, which preserved the brain atrophy pattern in MSA. We assume that alpha-synuclein oligomerization is involved in the neural cell death and brain atrophy. It might have caused further neural cell death in the brain damaged by hypoxia. Alpha-synuclein, which is involved in the pathogenesis of MSA, is suggested to be a prion. Misfolded alpha-synuclein may propagate through cell-to-cell transmission and cause wide pathological change, visible as atrophied MR imaging.

## Background

Multiple system atrophy (MSA) is a progressive neurodegenerative disease. MSA patients show phenotypes characterized by autonomic failure with motor involvement of parkinsonism and/or cerebellar ataxia. Autonomic dysfunction of MSA includes orthostatic hypotention, bowel and bladder disturbances, and erectile dysfunction in males (1, 2). MSA patients survive between 6.2 and 10 years, but some MSA patients survive for over 15 years (3). MSA comprises MSA-C (with cerebellar symptoms) and MSA-P (developing Parkinsonism). In MSA-C, the olivary nucleus, pons, and cerebellum cortex become atrophic. In MSA-P, the dorsolateral regions of both putamina are atrophic (4, 5). Several features of magnetic resonance imaging (MRI) have been identified in MSA. In MSA-P, there appears a bilateral T2-hyperintense rim, bordering the dorsolateral margins of the putamen (“hyperintense lateral putaminal rim”) and T2-putaminal hypointensity (6). In MSA-C, there appears a T2-hyperintense cross in the pons (“hot-cross-bun sign”) [reviewed in Ref. (7)]. MSA patients have narrower middle cerebellar peduncle and wider superior cerebellar peduncle compared to those of progressive supranuclear palsy (PSP) patients (8).

In MSA, mechanisms of neural cell death, which underlies brain atrophy, are unresolved. However, the involvement of alpha-synuclein in the neural cell death has been suggested by sizable number of evidences. Alpha-synuclein oligomers accumulate in the brain of the MSA patients to induce metabolic imbalance, which might promote neural cell death (9). Other mechanisms of the neural cell death in MSA include proteasomal or autophagosomal dysfunction and excitotoxicity [reviewed in Ref. (10)].

Brain MRIs of the patients developing hypoxic encephalopathy after cardiac arrest show different features. The diseased state and brain imaging findings vary and are influenced by age, baseline disease, and cardiac arrest period (11). In resuscitated and completely recovered patients, specific computed tomography (CT) imaging features are barely detectable. In the acute phase, the gray matter gives high-density signals on MRI (12). These signals increase in T2 and fluid-attenuated inversion recovery images by performing MRI after the subacute phase. By contrast, the signal intensity in the white matter rarely increases in such patients. Laminar necrosis in the cerebral cortex is often observed in hypoxic encephalopathy patients. Among hypoxic encephalopathy patients, those with hypoxic encephalopathy caused by cardiac arrest develop extensive global brain atrophy (12–16). It is well known that the gray matter is extensively damaged by hypoxic insult (12). However, whether the white matter is as severely damaged as the gray matter by hypoxic insult remains unknown.

## Case Presentation

A 65-year-old man developed Parkinsonism with resting tremor in his right hand, bradykinesia, and cerebellar dysarthria 10 years previously, from his death at 75 years of age in January 2017. He sometimes developed orthostatic hypotension. He also developed urinary incontinence with constipation. Postural reflex failure, finger-dexterity deficits, gait disturbance, and freezing of gait followed. He also developed irritability and memory deficit, but not cognitive impairment. When he previously visited a doctor in November 2008, no vertical gaze palsy had been noted and Babinski reflexes of both sides were observed. The early and delayed heart-to-mediastinum (H/M) uptake ratios on performing ^123^I-metaiodobenzylguanidine (MIBG) scintigraphy were 1.8 and 1.7, respectively (Ogura J: personal communication). His brothers, sisters, or children had no neurodegenerative disorders. He developed rigidity of the trunk and upper and lower limbs. Levodopa treatment (600 mg per a day) did not improve his Parkinsonism.

In August 2015, caregivers at the home for elderly people, where the patient resided, found him in cardiac arrest and transferred him to the hospital. When a doctor tried to intubate him, he found that the airway was obstructed by a piece of bread and removed it with Magill forceps. However, it took 18 min to find and remove the obstruction. The patient was intubated, received therapeutic hypothermia at 33°C, was rewarmed after 24 h, had recovered to 37°C by 48 h after starting hypothermia, and was then extubated. The corticomedullary junction was unclear in the CT scan, which suggested hypoxic encephalopathy. The patient was in mutative akinesia, and it was estimated that levodopa would not improve the disease; the amount of levodopa was reduced to 300 mg. Because of aggravation of dysphagia and bladder rectal disorders such as dysuria, he was admitted to our hospital in September 2015 in a bedridden state. At that time, he was fed liquid nutrition using nasal tube and could speak, but with cerebellar dysarthria. We observed the Babinski reflex and Chaddock reflex on both sides and the snout reflex. Orthostatic hypotension was also observed. He developed rigidity in the neck and upper and lower limbs. We increased the amount of levodopa up to 600 mg per day, but could not improve his Parkinsonism.

**Figure 1:**
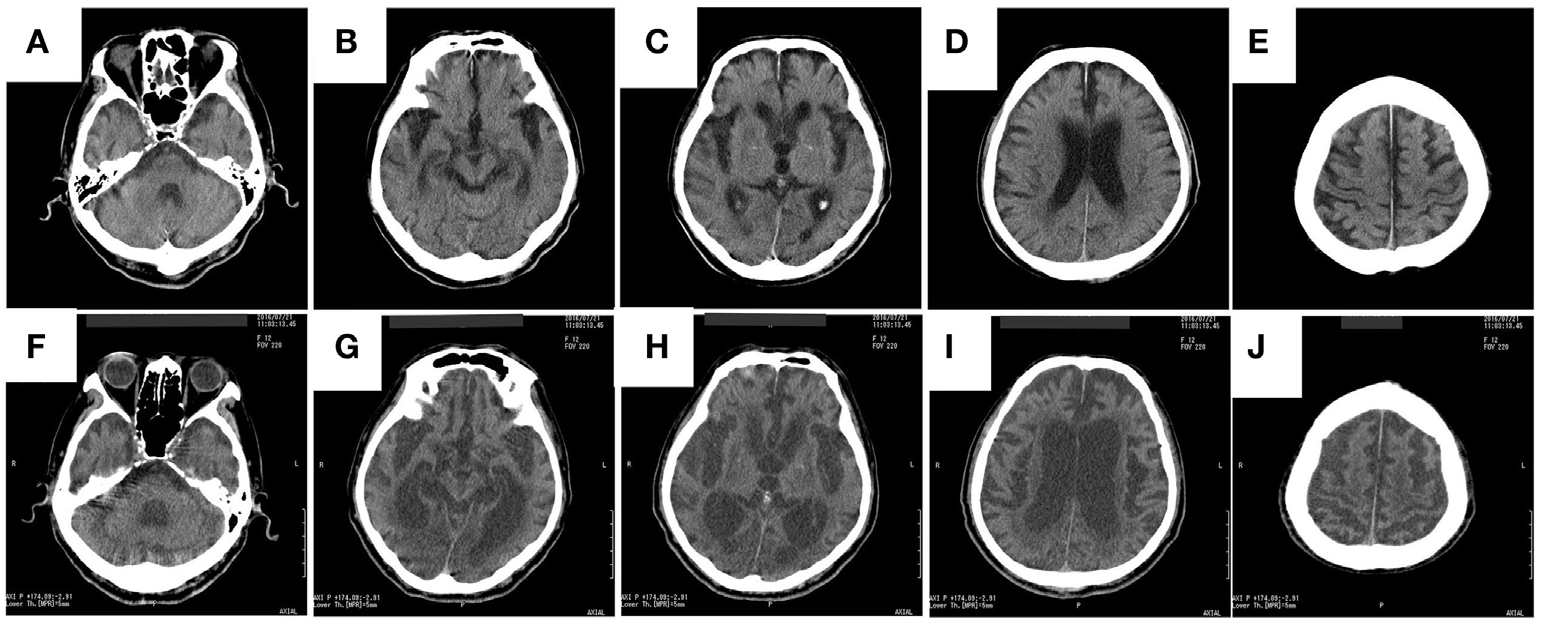
The rapid progress of brain atrophy in the MSA patient after developing hypoxic encephalopathy axial CT images of the patient acquired in September 2015 **(A–E)** and in July 2016 **(F–J)** are shown. MSA, multiple system atrophy; CT, computed tomography.

**Figure 2:**
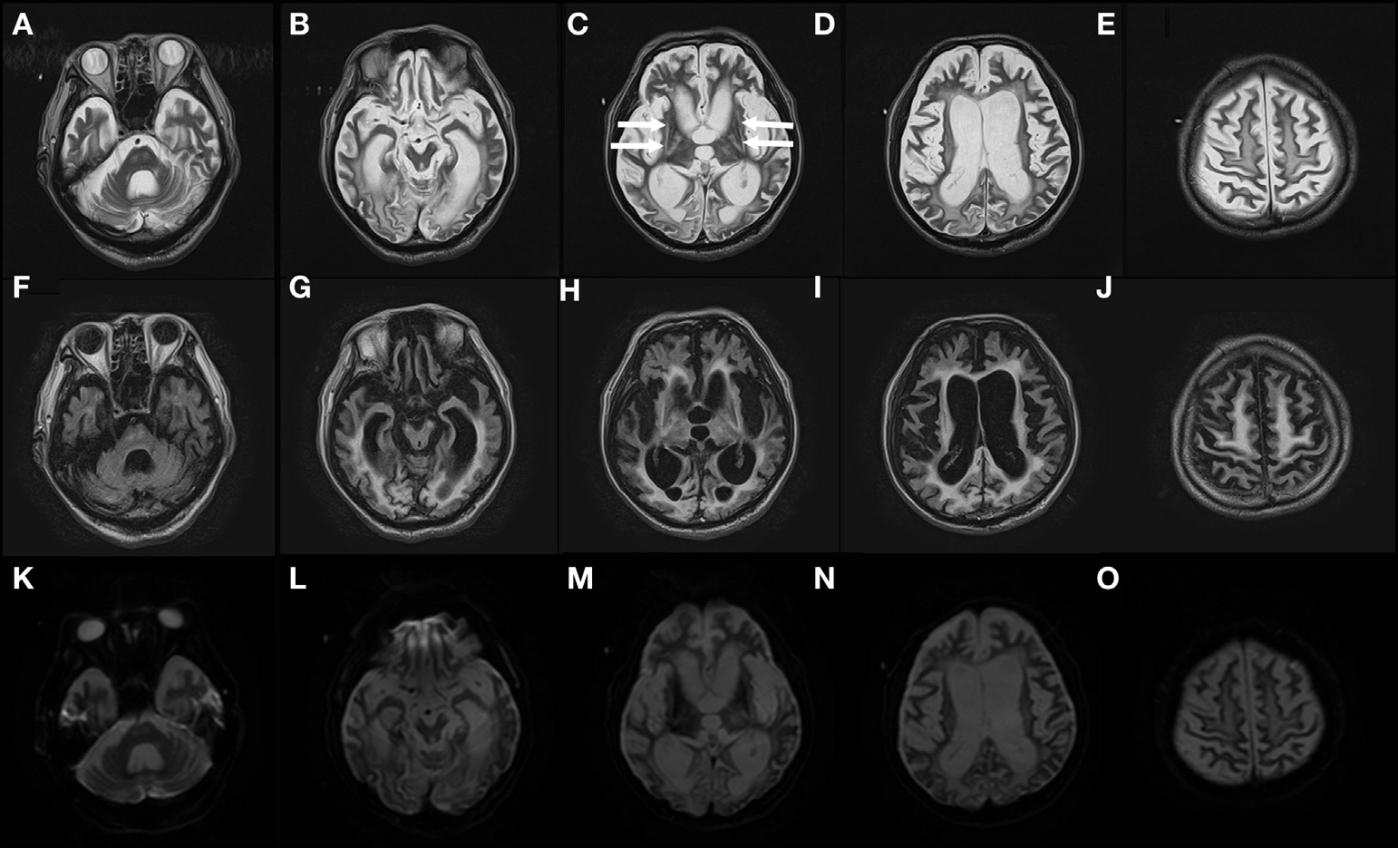
Severe atrophy in the MSA patient’s brain observed in August 2016 Gadolinium-enhanced MRI T2 images (TR: 4,800 ms, TE: 105 ms) **(A–E)**, fluid-attenuated inversion recovery images (TR 10,000 ms, TE 120 ms) **(F–J)**, and diffusion-weighted imaging images (TR 5,400 ms, TE 100 ms) **(K–O)** of the patient’s brain in August 2016 are shown. Arrows in **(C)** indicate dorsolateral regions of atrophy (“hyperintense lateral putaminal rim”). MSA, multiple system atrophy; MRI, magnetic resonance imaging.

**Figure 3:**
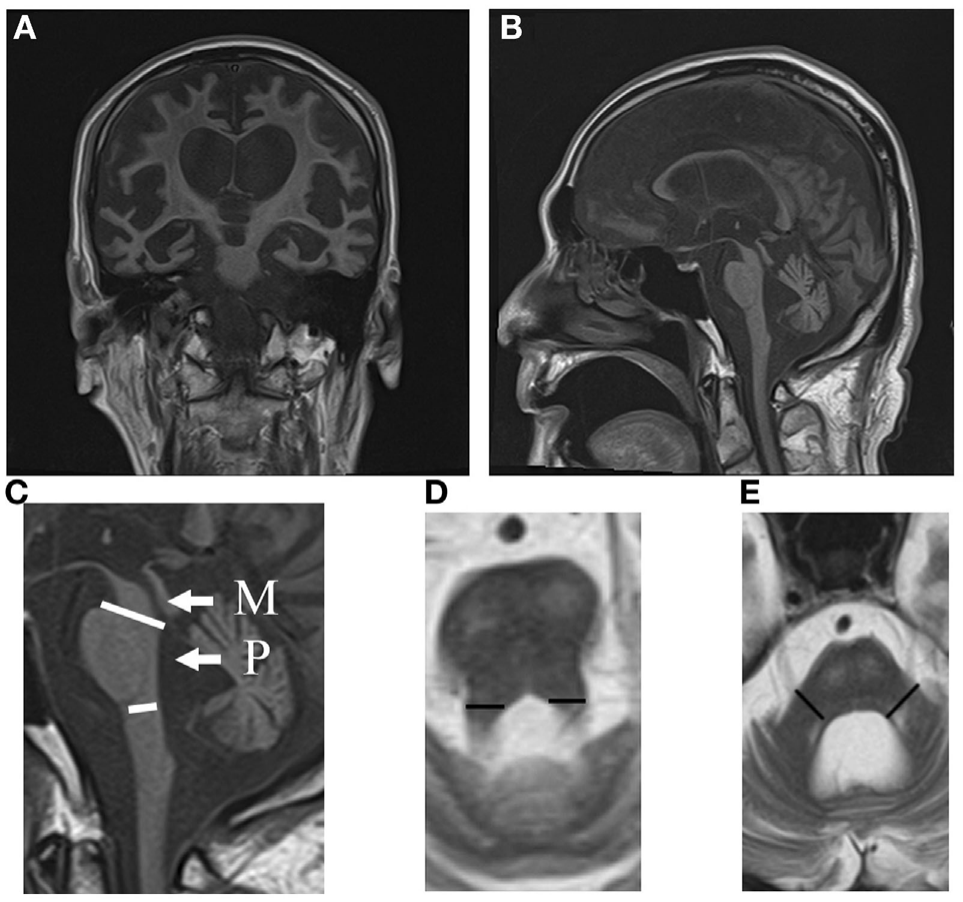
MRI coronal **(A)** and sagittal **(B)** T1 images (TR 540 ms, TE 15 ms) of the MSA patient in August 2016. Note the atrophy in the caudal pons. The midbrain and cerebellum are atrophic. **(C)** Areas of the midbrain (M) and pons (P) are shown. **(D)** The width of the superior cerebellar peduncles (segments) is shown. **(E)** The width of the middle cerebellar peduncles (segments) is shown. MSA, multiple system atrophy; MRI, magnetic resonance imaging.

Computed tomography assessment showed no apparent atrophy in the cortical region or brainstem in September 2015 (Figure 1). There was slight atrophy in the medial temporal lobe and slight dilatation of the lateral ventricles and Sylvian fissures. By contrast, marked atrophy of the cerebrum, brainstem, and cerebellum was observed by CT and MRI in July 2016 and after August 2016, respectively (Figures 1–3). Atrophy was observed in the midbrain, caudal pons, and striata on both sides (“hyperintense lateral putaminal rim”) (Figures 2 and 3), which strongly indicated MSA. The lateral, third, and fourth ventricles were markedly dilated. Measurement of the T1-weighted mid-sagittal image and T2-weighted axial images showed that the midbrain area and pons area were 75.3 and 321.2 mm^2^, respectively, and the mean widths of the superior cerebellar peduncles and middle cerebellar peduncles were 3.38 and 11.25 mm, respectively, as measured in the same manner as described in a previous study (8) (Figures 3C–E). Compared with the results in the previous report (8) (median area of the pons: 331 mm^2^; median width of the middle cerebellar peduncle, 11.7 mm; median width of the superior cerebellar peduncle, 3.3 mm), our results were consistent with the pattern of images associated with MSA, except for the highly atrophic midbrain, which might indicate the possibility of PSP. It should be noted that atrophy of the middle cerebellar peduncles, rather than the superior cerebellar peduncles, is a feature of MSA images.

**Figure 4:**
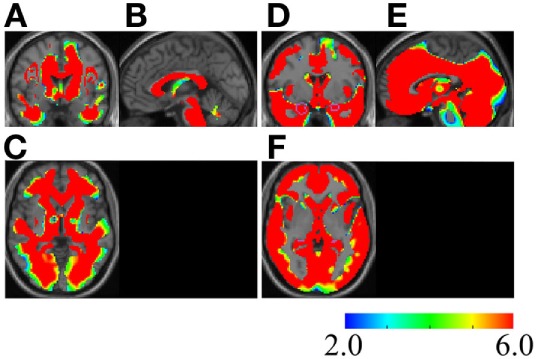
Severe atrophy in the white and gray matter of the MSA patient. *Z*-score maps of the white matter **(A–C)** and gray matter **(D–F)** of the MSA patient are shown. The brain image of the patient is compared with that of individuals without neurodegenerative diseases or dementia (17). The color scale for the *z*-score is shown on the bottom right. Colored areas indicating a *z*-score of >2 are overlaid as regions of significant atrophy. Coronal **(A,D)**, sagittal **(B,E)**, and axial **(C,F)** sections by magnetic resonance imaging (MRI) are presented on a standardized MRI template. Regions with a *z*-score of >6 exist widely in the patient’s brain, regardless of whether the region belongs to the white or gray matter. [Regions inside the purple polygons are medial temporal lobes, because the initial aim of the voxel-based specific regional analysis system for Alzheimer’s disease software creators was estimation of the atrophy rate of the area (17)]. MSA, multiple system atrophy.

Use of the voxel-based specific regional analysis system for Alzheimer’s disease (17–19) software according to the method used in previous studies on the same regions (18, 19) showed that the white and gray matter atrophy values were 77.36 and 80.49%, respectively, with a *z*-score >2 in October 2016 (Figure 4). From the time of admission, aspiration pneumonia occurred repeatedly and finally led to death in January 2017. At the request of the bereaved family, an autopsy was not performed.

## Discussion

The patient presented in this report had developed a sporadic, progressive, adult-onset disease characterized by poorly levodopa-responsive parkinsonism. He also developed autonomic failure, involving urinary incontinence and orthostatic hypotension, as well as cerebellar dysarthria. These symptomatic features fulfill the diagnostic criteria of “probable MSA” (4). He might belong to an atypical MSA, in that he lived 10 years after the onset of the disease. This might be partly due to our extensive care, nursing, and rehabilitation. Furthermore, a previous report described some MSA patients survived over 15 years (3). This diagnosis is supported by images showing, for example, atrophy in the dorsolateral regions of the putamina (Figures 2C,H), and atrophy in the cerebellum and caudal regions of the pons (Figure 3B).

Our patient exhibited moderate decrease in uptake of MIBG, whereas a marked decrease in MIBG uptake in patients with Parkinson disease and those who have dementia with Lewy bodies has been reported in many studies. The moderate reduction in uptake by our patient supports a diagnosis of MSA, as suggested by some previous studies (20–22). Literature on the H/M ratios of MSA, PSP, Parkinson disease, and healthy controls in MIBG scintigraphy has been reviewed by Chung and Kim (23). We searched the literatures in their review, and calculated mean ± SD using the representative value and N from each literature, and compared them with our H/M ratios (early, 1.8; late, 1.7) and examined the estimated *p*-values, provided that the data were from normally distributed data. H/M ratios of the patient are more likely to be those in MSA (early: *p* = 0.175 > 0.1; delayed: *p* = 0.137 > 0.1) rather than those in Parkinson disease (early: *p* < 0.1; delayed: *p* < 0.01) nor PSP (early: *p* < 0.05; delayed: *p* < 0.05). The data of the patient declined from those of the normal control (early: *p* < 0.1; delayed: *p* < 0.01).

Next, we performed a differential diagnosis by considering other diseases with symptoms resembling MSA-P. Parkinson disease was excluded because Parkinsonism of the patient responded poorly to 600 mg levodopa per day. Creutzfeldt–Jakob disease (CJD) was also excluded because diffusion-weighted imaging gave no sign of high intensity, which was typical for images of CJD. Corticobasal degeneration was excluded because the patient did not show cortex symptoms that matched the diagnostic criteria (24). The symptoms of the patient did not fit the one major criteria of probable PSP: vertical gaze palsy (25) although previous reports have suggested that 10–50% of the patients diagnosed with PSP did not develop vertical gaze palsy (25).

A limitation of this study was that we could not obtain permission for an autopsy, so we could only provide a probable diagnosis.

Progression of symptoms, pathological aspects, and MSA brain imaging findings of longitudinal studies have been reported. Whole-brain atrophy rates in MSA have been 1.65 ± 1.12 or 1.65 ± 0.9% per year (5, 26). These results are in contrast to our present results showing that 77.36 and 80.49% of the patient’s white and gray matter had become atrophic within 1 year. This discrepancy is probably because of the influence of hypoxia synergizing with neural cell death mechanisms in MSA.

The brain region susceptible to hypoxia is supposed to be the gray matter (12). Some studies have detected white matter damage in the early stage of hypoxic encephalopathy (13–15). In our resuscitated MSA patient, the white matter was damaged almost as severely as the gray matter.

We propose a hypothesis as to why the extensive brain atrophy progressed so rapidly within 10 months, whereas we observed only a slight atrophy 1 month after cardiac arrest and resuscitation. Hypoxic insult triggers alpha-synuclein oligomerization (27). Once the aggregation of alpha-synuclein oligomers begins, the aggregation accelerates and proceeds very rapidly (28). Alpha-synuclein is a cytotoxic (29), and expanded alpha-synuclein oligomers might induce neural cell death, manifesting through MRI or CT as brain atrophy. Hypotoxic–ischemic insult might be in itself harmful to neural cells. Alpha-synuclein oligomers might aggravate brain atrophy by their cytotoxicity.

In MSA, glial cytoplasmic inclusion (GCI) shows up mainly in the white matter; however, neuronal cytoplasmic inclusion (NCI) can also be seen in the gray matter (30). Hypoxia-induced misfolded alpha-synucleins accumulate to form GCI and NCI in the white and gray matter, respectively, and might induce neurodegeneration in each region (31, 32).

An alternative hypothesis on the rapid and extensive brain atrophy of the MSA patient comes from the fact that atrophic brain images at the end stages of MSA patients are reminiscent of the brain images of patients with prion diseases such as CJD. MSA is a synucleinopathy, and alpha-synuclein is regarded as a prion (33, 34). In MSA patients, aggregation of misfolded alpha-synuclein might cause endoplasmic reticulum (ER) stress, which results in nerve cell apoptosis (31, 35). Hypoxia, another ER stress inducer (31, 35), enhances ER stress and nerve cell apoptosis triggered by toxic alpha-synuclein. Misfolded alpha-synuclein spreads through cell-to-cell transmission to cause wide pathological changes (36, 37). Extensive brain atrophy might be a manifestation of such pathological changes. Thus, the brain of an MSA patient might develop rapid and severe atrophy under hypoxia in the way that brains with prion diseases do, possibly by common mechanisms.

## Ethics Statement

Approved by the Hospital Ethics Committee. Written permission was obtained from the patient’s next of kin.

## Author Contributions

SN, HM, and YK planned and designed the study; SN, HM, and YK acquired and analyzed the data; and SN wrote the manuscript. All authors approved submission of the manuscript.

## Conflict of Interest Statement

The authors declare that the research was conducted in the absence of any commercial or financial relationships that could be construed as a potential conflict of interest.

## Abbreviations

CJD, Creutzfeldt–Jakob disease; CT, computed tomography; DWI, diffusion-weighted image; ER, endoplasmic reticulum; FLAIR, fluid-attenuated inversion recovery; GCI, glial cytoplasmic inclusion; H/M, heart-to-mediastinum; MIBG, metaiodobenzylguanidine; MRI, magnetic resonance imaging; MSA, multiple system atrophy; NCI, neuronal cytoplasmic inclusion; PSP, progressive supranuclear palsy; VSRAD, voxel-based specific regional analysis system for Alzheimer’s disease.
